# Survey data on digitalization of building procurement process by architectural firms in Abuja, Nigeria

**DOI:** 10.1016/j.dib.2018.08.187

**Published:** 2018-09-05

**Authors:** Eziyi O. Ibem, Adedotun O. Akinola, Emokpae M. Erebor, Morolaoluwa O. Tolani, Adaeze E. Nwa-uwa

**Affiliations:** Department of Architecture, Covenant University, Canaanland, Ota, Ogun State, Nigeria

**Keywords:** Digital technologies, Building procurement, Architectural firms, Survey, Abuja

## Abstract

This data article describes the dataset on the digital technologies and applications used by architectural firms in the delivery of building projects. The data set was sourced through a questionnaire survey of 75 registered architectural firms in Abuja, Nigeria. The survey was conducted between December 2017 and January 2018 in the study area. The data, which were analyzed using descriptive statistics, contain the characteristics of architectural firms in Abuja that participated in the survey, the different stand-alone and integrated data acquisition, creation, processing, storage and communication/exchange technologies, applications and tools and intelligent systems the firms use in building procurement process. In addition, the advantages and challenges associated with the use of digital technologies in the procurement of building projects by architectural firms in the study area are also presented in this dataset.

**Specification table**

**Table:** 

Subject area	Architecture and Construction
More specific subject area	Information technology adoption in architecture and construction
Type of data	Tables and Figures
How data was acquired	Survey
Data format	Raw and analysed
Experimental factors	Cross-sectional survey of registered architectural firms in Abuja. The architectural firms and respondents were selected using a combination of purposive and random sampling techniques.
Experimental features	Only one respondent in each architectural firm participated in the survey. Stand alone and integrated digital technologies used to support the execution of intra and inter-firms building procurement activities were investigated.
Data source location	Abuja, Nigeria
Data accessibility	Both the proposed data and analysed results are shared in this article

**Value of the data**

• The dataset is valuable as it contains information on the key aspect of building procurement process that lends itself most to the application of digital technologies and tools in architectural practice in the study area.
• The dataset is also valuable in revealing the benefits and challenges that come with the digitalization of building procurement process by architectural firms, and thus provides insight into the e-readiness of architectural firms in a developing country like Nigeria.
• The data shared in this article can contribute to further research on the possible strategies for overcoming the challenges that come with the use of digital technologies in the delivery of building projects by architects in Nigeria.
• The dataset can be used to benchmark architectural firms in Nigeria with their counterparts in other countries on the use of digital technologies, tools, and processes.
• Other researchers on this subject can also derive some benefits from the questionnaire instrument shared via this article.

## 1 Data

In this article, three data files are shared. These are the questionnaire file, the Statistical Package for the Social Sciences (SPSS) file and the file containing Tables and Figures. The questionnaire file contains the research instrument used in the extraction of the dataset from participants in the survey. The questionnaire instrument contains a set of questions describing the characteristics of the architectural firms such as their ownership structure, age, the number of employees, number of offices in Nigeria, the different types of professionals services rendered, by them, categories of clients the firms renders services to, ownership of functional website and their access to the Internet services. The characteristics of the firms that participated in the survey are displayed in Table 1.

**Table 1:** Characteristics of firms included in the survey.

**Characteristics of firms**	**Frequency (*n* = 75)**	**Percent (% = 100)**
**Ownership structure**		
Sole proprietorship	24	32.0
Partnership	33	44.0
Limited liability company	15	20.0
Consortium	3	4.0
**Age in years**		
No response	1	1.3
Less than 5 years	22	29.3
6–10	31	41.3
11–15	13	17.3
16+	8	10.7
**Number of employees**		
No Response	2	2.7
Below 5 Persons	22	29.3
5–10 Persons	32	42.7
11 Persons and above	19	25.3
**Number of offices in Nigeria**		
1	37	49.3
2	25	33.3
3	9	12.0
More than 3 offices	4	5.3
**Types of professional services rendered**		
No Response	1	1.3
Design only	20	26.7
Design and Build	46	61.3
Project Management	8	10.7
**Categories of clients**		
Government	24	32.0
Corporate private organisations	17	22.7
Individuals	26	34.7
Non-Governmental Organisations	5	6.7
Religious organisations	3	3.9
**Ownership of functional website**		
Yes	50	67.0
No	25	33.0
**Regular access to the Internet**		
Yes	72	96.0
No	3	4.0

Although previous studies [1], [2], [3] have investigated architectural firms in Nigeria, none of them attempted to examine the extent of digitalisation of the building procurement process by registered firms. Moreover, a previous study [4] has identified the various applications of digital technologies, applications, and tools in the procurement of construction projects via a systematic review of research literature. In view of the foregoing, and in addition to the characteristics of the firms, the questionnaire instrument also had questions on the various digital technologies, applications and tools the firms use in the acquisition, creation, processing, storage, display, and communication/exchange of building project data and information and the intelligent systems used in the integration of people, activities/tasks, and processes involved in all stages in the delivery of building projects. There are also questions on the social, economic, and technological advantages and challenges that come with the use of these technologies in the building procurement process. The SPSS file contains the processed data showing the different variables, their scales of measurement and coding of the responses provided to the questions in the questionnaire by the participants in the survey. This file contains the descriptions of all the 79 variables investigated in the research.

The Tables and Figures file contains the descriptive statistics of the responses obtained via the questionnaire administered to the 75 firms that participated in the survey in the study area. The responses are based on their experience with the use of the different categories of digital technologies and applications in the delivery of building projects. This file contains three categories of data. The first category is the distribution of the principals or representatives of the firms (who are the respondents in the survey) according to their highest level of educational attainment. Second are the participants’ responses to the questions on the frequency of use of the different data acquisition, creation, processing, storage, display, and communication/exchange technologies, applications and tools as well as the intelligent systems used to execute building procurement activities as identified in the questionnaire. The responses are presented using frequencies distribution and percentages. The last category of data includes the responses to the questions on 17 advantages and 11 challenges associated with the used the various types of digital technologies in the delivery of building projects. In addition to presenting the responses using frequencies and percentages, mean scores and ranking of mean scores were also used for a better understanding of the data.

## 2 Experimental design, materials, and methods

The dataset shared on this article was created using a cross-sectional survey conducted between December 2017 and January 2018 in Abuja, Nigeria. Abuja was chosen for the survey because it is the capital city of Nigeria and has a large proportion of registered architectural firms in this country outside Lagos. The survey involved the administration of questionnaire to the participants directly involved in the design, tendering, and construction phases of building projects in the selected firms. Several previous studies [5], [6], [7], [8] have adopted this approach in extracting data from stakeholders on various research issues. In order to ensure that the dataset was derived from adequate number of registered architectural firms involved in building procurement activities in the study area, the formula developed by Yamane [9] for finite population was used in the determination of the sample size for the survey. The formulation is given as:

$$n=\frac{N}{{1+N(e}^{2})}$$

In using this formula, n represents the sample size (i.e. number of people to be selected); *N* is the size of the population that sample could be drawn from, while *e* is the assumed level precision, which is ± 5% (*e* = 0.05) at 95% confidence level. From the list of registered architectural firms licensed to practice in Nigeria published by the Architects Registration Council of Nigeria (ARCON) in 2016, it was found that 190 of these firms have office in Abuja. Therefore, using *N* = 190 the sample size for this survey was computed as follows

*n* = 190/1 + 190 (0.05^2^)
*n* = 190/1 + 190(0.0025)
*n* = 190/1.475
*n* = 128.8 = 129 firms

This means that the sample size for the survey was 129 registered firms.

The questionnaire used in generating the dataset consists of four main sections: A, B, C and D. Section A deals with the characteristics of the firms from where the participants were selected, while section B had questions on the frequency of use of the various stand-alone and integrated data acquisition, creation, processing, storage and communication/exchange technologies, applications and tools as well as the intelligent systems used in the design, tendering and construction of building projects by the firms. The scale of measurement used in section B was 3-point Likert type scale: “1” for *Never Used*, “2” for *Used Sometimes*, and “3” for *Used Always*. Previous studies [10], [11] have also used this scale The third section (Section C) had questions on the advantages that come with the use of the aforementioned categories of digital technologies in the delivery of building projects, while the fourth section (Section D) was used to collect data on the challenges experienced with the use of digital technologies in the delivery of building projects. In these last two sections of the questionnaire, the scale of measurement used was 5-point Likert type scale ranging from “1” for *Strongly Disagree*, to “5” for *Strongly Agree*. Other authors have used the aforementioned scale of measurement in previous research works [11], [12], [13]. At the pre-testing stage of the questionnaire, Cronbach׳s Alpha test was used to investigate the reliability of this scale of measurement used for the 17 items in Section C and 11 items in Section D. The tests returned values: 0.942 and 0.753 for the items in Sections C and D, respectively. These values are higher than the acceptable value of 0.7 recommended in the literature [14]. A sample copy of the questionnaire used in extracting the raw data from the respondents in the survey is included in the supplementary file associated with this article.

Based on the calculated sample size, 129 copies of the questionnaire were administered by hand to randomly selected firms in the study area. Specifically, one questionnaire was given to the head (principal) of each firm or his/her appointed representative only. This was to ensure that the data was sourced from persons with experience in building procurement activities in the firms sampled. However, 75 copies representing around 58% of the questionnaires administered were retrieved by hand. The data from the 75 questionnaires retrieved were processed using computer and subsequently analyzed with the help of the SPSS. The data were analysed using descriptive statistics including, frequencies, and percentages, mean scores, standard deviations, and ranking. Specifically, mean scores and ranking were used in the analysis of the data on advantages and challenges associated with the use of digital technologies and applications in the delivery of building projects. Further details of the results of the analysis presented using tables and charts are shown in the supplementary files associated with this data article.

## References

[1] Oluwatayo A.A., Amole A.D. (2012). Characteristics of global architecture firms. Eng. Constr. Arch. Manag..

[2] Oluwatayo A.A., Amole A.D. (2013). Ownership, structure and performance of architecture firms. Front. Arch. Res..

[3] Oluwatayo A.A., Amole D. (2014). Organizational structure of architectural firms and their performances. Int. J. Constr. Eng. Manag..

[4] Ibem E.O., Laryea S. (2014). A survey of digital technologies in procurement of construction projects. Autom. Constr..

[5] Ibem E.O., Aduwo E.B., Uwakonye U.O., Tunji-Olayeni P.F., Ayo-Vaughan E.A. (2018). Survey data on e-procurement adoption in the nigerian building industry. Data Brief.

[6] Iroham C.O., Okagbue H.I., Ogunkoya O.A., Owolabi J.D. (2017). Survey data on factors affecting negotiation of professional fees between estate valuers and their clients when the mortgage is financed by bank loan: a case study of mortgage valuations in Ikeja, Lagos State, Nigeria. Data Brief.

[7] Amusan L.M., Afolabi A., Ojelabi R., Omuh I., Okagbue H.I. (2018). Data exploration on factors that influences construction cost and time performance on construction project sites. Data Brief.

[8] Afolabi A.O., Ojelabi R.A., Tunji-Olayeni P.F., Fagbenle O.I., Mosaku T.O. (2018). Survey datasets on Women participation in Green jobs in the Construction Industry. Data Brief.

[9] Yamane, T T. (1967). Statistics: An Introductory Analysis.

[10] Aduwo E.B., Ibem E.O., Uwakonye U.O., Ayo-Vaughan E.A., Owolabi J. (2017). E-procurement use in the nigerian building industry. Int. J. Electron. Commer. Stud..

[11] Ibem E.O., Aduwo E.B., Ayo-Vaughan E.K., Tunji-Olayeni P.F. (2018). A survey of digital technologies used in the procurement of building projects: empirical evidence from Nigeria. Asian J. Sci. Res..

[12] Ibem E.O., Aduwo E.B., Ayo-Vaughan E.K. (2017). E-Procurement Adoption in the Nigerian Building Industry: architects perspective. Pollack Period.- Int. J. Eng. Inf. Sci..

[13] Ibem E.O., Aduwo E.B., Tunji-Olayeni P., Uwakonye U.O., Ayo-Vaughan, E. K E.K. (2016). Factors affecting E-procurement adoption in the nigerian building industry. Constr. Econ. Build..

[14] Pallant J. (2011). SPSS Survival Manual-a Step by Step Guide to Data Analysis using SPSS.

