# Supplementary material for: Survey data on digitalization of building procurement process by architectural firms in Abuja, Nigeria
Source: Data Brief. 2018 Sep 5;20:1062–7. doi: 10.1016/j.dib.2018.08.187 (PMC6139998; doi:10.1016/j.dib.2018.08.187)
Supplement: Supplementary file 1 — Transparency document. [file mmc1.doc]

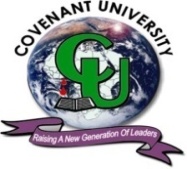


Eziyi O. Ibem; *Ph.D (Architecture); M.Sc (Arch); B.Sc (Arch) mnia, mnehr*

*Professor of Architecture*

Department of Architecture, Covenant University, Ota, Ogun State, Nigeria

[ibem.eziyi@covenantuniversity.edu.ng](mailto:ibem.eziyi@covenantuniversity.edu.ng) ; [eziyioffia@yahoo.com](mailto:eziyioffia@yahoo.com).

+234(0)8037779415; 08189892900

6 August 2018

**Editor, Data in Brief**

**Conflict of Interest**

This is to declare that there is no conflict of interest among the authors of the manuscript entitled ‘Survey *Data on Digitalisation of Building Procurement Process by Architectural Firms in Abuja, Nigeria*’ submitted for consideration in Data in Brief*.*

The authors have read the final draft and agreed that the manuscript be sent for review in this journal.

Thank you.


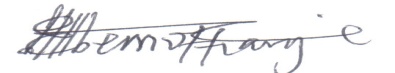


**Prof. Eziyi O. Ibem**; *Ph.D;*

*Corresponding Author*
