# Supplementary material for: Survey data on digitalization of building procurement process by architectural firms in Abuja, Nigeria
Source: Data Brief. 2018 Sep 5;20:1062–7. doi: 10.1016/j.dib.2018.08.187 (PMC6139998; doi:10.1016/j.dib.2018.08.187)
Supplement: Supplementary file 2 — Supplementary material. [file mmc2.zip › Questionnaire.doc]

**Covenant University, Ota, Ogun State Nigeria**

**Questionnaire**

Dear Respondent,

The research seeks to identify the digital technologies used in the delivery of building projects by architectural firms in Abuja, Nigeria. All information provided will be treated with strict anonymity and used only for academic purposes.

Thank you for engaging with us in this research.

**Please tick (√) as appropriate**

**A: Basic Information**

1. Please indicate the type of your firm. Sole Proprietorship [	] Partnership [	] Limited Liability Company [	]

Consortium [	]

2. How old is your firm? Less than 5 years [	] 6 years-10years [	] 11-15 years [	]	16 years + [	]
3. The number of permanent employees in your firm is Below 5 persons [	] 5-10 Persons [ ] 11 persons +[	]
4. How many offices do you have in Nigeria? 1 [	] 2 [	] 3 [	]More than 3 offices [	]

5. Which of these categories of client patronize your firm most? Government [	] Individuals [	] Private Corporate organisations [	] Religious Organisations [	] Non-governmental organisations [	]
6. Which of these professional services do you firm render to clients? Design only [	] Design and Build [	]

Project Management [ ] Others, please specify--------------------------------------------------------------

7. Does your firm have a functional website? Yes [	]	No [	]
8. Do you have regular access to Internet services in your offices? Yes [	]	No [	]
9. What is your highest educational qualification? ND [	] HND [	] B.Sc [	] M.Sc [	] Ph.D [	]

**B: Digital technologies used in project delivery**

|  | **Please indicate how often you use these digital technologies in the delivery of projects in your firm** | *Never used* | *Used Sometimes* | *Used Always* |
| --- | --- | --- | --- | --- |
| 1 | **Data acquisition technologies** |  |  |  |
| a | Multimedia technology |  |  |  |
| b | Radio Frequency Identification (RFID) |  |  |  |
| c | Bar code technology |  |  |  |
| d | Project camera |  |  |  |
| e | Internet-Based Geographic information System (GIS) |  |  |  |
| f | Geographic positioning system (GPS) |  |  |  |
| g | Sensor Network |  |  |  |
| 2 | **Data creation, processing and storage applications** |  |  |  |
| a | Computers |  |  |  |
| b | Servers |  |  |  |
| c | Word process software packages |  |  |  |
| d | Computer aided design (CAD) software packages |  |  |  |
| e | Cost estimation and analysis software packages |  |  |  |
| f | Hard discs, |  |  |  |
| g | RAM discs |  |  |  |
| h | Optical discs |  |  |  |
| i | Smart/ memory cards |  |  |  |
| j | Flash drives |  |  |  |
| k | Compact disc |  |  |  |
| l | Compact Disc Read-Only Memory (CD-ROM) |  |  |  |
| **3** | **Data communication/exchange technologies** |  |  |  |
| a | Video conferencing technology |  |  |  |
| b | Tele conferencing technology |  |  |  |
| c | Wireless Technology (WT) |  |  |  |
| d | Electronic Data Interchange (EDI) |  |  |  |
| e | e-Mail |  |  |  |
| f | Electronic Data Exchange (EDI) |  |  |  |
| g | Network technologies |  |  |  |
| h | Web.2.0 technology ( e.g. social media platforms) |  |  |  |
| i | Telephone |  |  |  |
| j | Fax |  |  |  |
| k | Voice of Internet Protocol ( e.g. Skype |  |  |  |
| l | Dropbox |  |  |  |
| m | Microsoft Sharepoint |  |  |  |
| n | Google documents |  |  |  |
| **4** | **Intelligent systems** |  |  |  |
| a | Web-based Project management software packages |  |  |  |
| b | Building Information Modeling (BIM) |  |  |  |
| c | Web-based Project Portals |  |  |  |
| d | Cloud computing technology (CCT) |  |  |  |
| e | e-Marketplaces |  |  |  |
| f | Web-based Project Portals |  |  |  |
| g | Web-based Enterprise Resource Planning |  |  |  |
| h | 3-D printer |  |  |  |
| i | Virtual Reality Technology (VRT) |  |  |  |

**C: Advantages in the use of digital technologies**

| **Please indicate the extent to which you agree with the following statements as it relate to the benefits of using digital technologies in project delivery [***1= “Strongly Disagree”; 2 = “Disagree”; 3= “Undecided” 4 = “Agree” 5 = “Strongly Agree”]* | | | | | | |
| --- | --- | --- | --- | --- | --- | --- |
|  | **The use of digital technologies** | **1** | **2** | **3** | **4** | **5** |
| 1 | Allows for access to centrally stored information |  |  |  |  |  |
| 2 | Saves time |  |  |  |  |  |
| 3 | Saves cost in project delivery |  |  |  |  |  |
| 4 | Reduces the use of paper work |  |  |  |  |  |
| 5 | Makes it easier exchange of project information |  |  |  |  |  |
| 6 | Facilitates the reduction of waste in project delivery |  |  |  |  |  |
| 7 | Helps in monitoring project site and collect real-time data |  |  |  |  |  |
| 8 | Promotes collaborative work |  |  |  |  |  |
| 9 | Helps in inventory management and record keeping |  |  |  |  |  |
| 10 | Promotes integration of tasks among consultants |  |  |  |  |  |
| 11 | Automates project delivery process |  |  |  |  |  |
| 12 | Promotes efficiency in project delivery |  |  |  |  |  |
| 13 | Improves productivity of firms |  |  |  |  |  |
| 14 | Is less labour intensive |  |  |  |  |  |
| 15 | Elimination of geographic barrier in project execution |  |  |  |  |  |
| 16 | Reduces errors |  |  |  |  |  |
| 17 | Facilitates quick decision making |  |  |  |  |  |

**D: Challenges in the use of digital technologies**

| **Please indicate the extent you agree with the following statements as they relate to the challenges associated with the use of digital technologies in project [***1= “Strongly Disagree”; 2 = “Disagree”; 3= “Undecided” 4 = “Agree” 5 = “Strongly Agree”]* | | | | | | |
| --- | --- | --- | --- | --- | --- | --- |
|  |  | **1** | **2** | **3** | **4** | **5** |
| 1 | Digital technologies are expensive to acquire and use |  |  |  |  |  |
| 2 | There is insecurity of project data with digital technologies |  |  |  |  |  |
| 3 | There is a lack of confidentiality of project data |  |  |  |  |  |
| 4 | There is the problem of incompatibility of different applications |  |  |  |  |  |
| 5 | Some digital technologies are not available in Nigeria |  |  |  |  |  |
| 6 | Special training is required before one can use digital technologies |  |  |  |  |  |
| 7 | Many people in the building industry do not know about digital technologies |  |  |  |  |  |
| 8 | There are delays in the transmission of data and information via the Internet |  |  |  |  |  |
| 9 | Some digital technologies are not user friendly |  |  |  |  |  |
| 10 | Digital technologies does not encourage creativity in architectural design |  |  |  |  |  |
| 11 | The use of digital technologies can lead to job loss in the industry |  |  |  |  |  |
