# Supplementary material for: Survey data on digitalization of building procurement process by architectural firms in Abuja, Nigeria
Source: Data Brief. 2018 Sep 5;20:1062–7. doi: 10.1016/j.dib.2018.08.187 (PMC6139998; doi:10.1016/j.dib.2018.08.187)
Supplement: Supplementary file 2 — Supplementary material. [file mmc2.zip › Table and figures .doc]

.

**Figure 1: Highest level of educational qualification of the respondents**

***Frequency of use of the digital technologies used in design of building projects***

.

**Figure 2: frequency of use of	Computer Aided Design (CAD)**

**Table 1:** Cost Estimation and Analysis Software Packages

| **Frequency of Use** | **Frequency** | **Percent** | **Valid Percent** | **Cumulative Percent** |
| --- | --- | --- | --- | --- |
| Never Used | 10 | 13.3 | 13.3 | 13.3 |
| Used Sometimes | 17 | 22.7 | 22.7 | 36.0 |
| Used Always | 48 | 64.0 | 64.0 | 100.0 |
| Total | 75 | 100.0 | 100.0 |  |

**Table 2:** Virtual Reality Technology (VRT)

| **Frequency of Use** | **Frequency** | **Percent** | **Valid Percent** | **Cumulative Percent** |
| --- | --- | --- | --- | --- |
| **Never Used** | 30 | 40.0 | 40.0 | 40.0 |
| **Used Sometimes** | 31 | 41.3 | 41.3 | 81.3 |
| **Used Always** | 14 | 18.7 | 18.7 | 100.0 |
| **Total** | 75 | 100.0 | 100.0 |  |

**Table 3:** Video conferencing technology

| **Frequency of Use** | **Frequency** | **Percent** | **Valid Percent** | **Cumulative Percent** |
| --- | --- | --- | --- | --- |
| **Never Used** | 16 | 21.3 | 21.3 | 21.3 |
| **Used Sometimes** | 29 | 38.7 | 38.7 | 60.0 |
| **Used Always** | 30 | 40.0 | 40.0 | 100.0 |
| Total | 75 | 100.0 | 100.0 |  |

**Table 4: Sensor Network**

| **Frequency of Use** | **Frequency** | **Percent** | **Valid Percent** | **Cumulative Percent** |
| --- | --- | --- | --- | --- |
| **Never Used** | 30 | 40.0 | 40.0 | 40.0 |
| **Used Sometimes** | 28 | 37.3 | 37.3 | 77.3 |
| **Used Always** | 17 | 22.7 | 22.7 | 100.0 |
| **Total** | 75 | 100.0 | 100.0 |  |

**Table 5**: Multimedia Technology

| **Frequency of Use** | **Frequency** | **Percent** | **Valid Percent** | **Cumulative Percent** |
| --- | --- | --- | --- | --- |
| **Never Used** | 8 | 10.7 | 10.7 | 10.7 |
| **Used Sometimes** | 27 | 36.0 | 36.0 | 46.7 |
| **Used Always** | 40 | 53.3 | 53.3 | 100.0 |
| **Total** | 75 | 100.0 | 100.0 |  |

**Figure 3: Word Process software packages**

**Table 6: The use of Servers**

| Frequency of Use | **Frequency** | **Percent** | **Valid Percent** | **Cumulative Percent** |
| --- | --- | --- | --- | --- |
| **Never Used** | 5 | 6.7 | 6.7 | 6.7 |
| **Used Sometimes** | 21 | 28.0 | 28.0 | 34.7 |
| **Used Always** | 49 | 65.3 | 65.3 | 100.0 |
| **Total** | 75 | 100.0 | 100.0 |  |

**Table 7: The use of** Hard Discs

|  | **Frequency** | **Percent** | **Valid Percent** | **Cumulative Percent** | |
| --- | --- | --- | --- | --- | --- |
| **Never Used** | 13 | 17.3 | 17.3 | 17.3 |  |
| **Used Sometimes** | 20 | 26.7 | 26.7 | 44.0 |  |
| **Used Always** | 42 | 56.0 | 56.0 | 100.0 |  |
| **Total** | 75 | 100.0 | 100.0 |  |  |

**Table 8: The use** RAM Discs

| **Frequency of Use** | **Frequency** | **Percent** | **Valid Percent** | **Cumulative Percent** |
| --- | --- | --- | --- | --- |
| **Never Used** | 25 | 33.3 | 33.3 | 33.3 |
| **Used Sometimes** | 34 | 45.3 | 45.3 | 78.7 |
| **Used Always** | 16 | 21.3 | 21.3 | 100.0 |
| **Total** | 75 | 100.0 | 100.0 |  |

**Table 9: The use of Optical Discs**

| **Frequency of Use** | **Frequency** | **Percent** | **Valid Percent** | **Cumulative Percent** |
| --- | --- | --- | --- | --- |
| **Never Used** | 27 | 36.0 | 36.0 | 36.0 |
| **Used Sometimes** | 38 | 50.7 | 50.7 | 86.7 |
| **Used Always** | 10 | 13.3 | 13.3 | 100.0 |
| **Total** | 75 | 100.0 | 100.0 |  |

**Table 10: The use of Smart/Memory cards**

| **Frequency of Use** | **Frequency** | **Percent** | **Valid Percent** | **Cumulative Percent** |
| --- | --- | --- | --- | --- |
| **Never Used** | 8 | 10.7 | 10.7 | 10.7 |
| **Used Sometimes** | 33 | 44.0 | 44.0 | 54.7 |
| **Used Always** | 34 | 45.3 | 45.3 | 100.0 |
| **Total** | 75 | 100.0 | 100.0 |  |

.

**Table 11: Flash Drives**

| **Frequency of Use** | **Frequency** | **Percent** | **Valid Percent** | **Cumulative Percent** |
| --- | --- | --- | --- | --- |
| Never Used | 3 | 4.0 | 4.0 | 4.0 |
| Used Sometimes | 16 | 21.3 | 21.3 | 25.3 |
| Used Always | 56 | 74.7 | 74.7 | 100.0 |
| Total | 75 | 100.0 | 100.0 |  |

**Table 12**: Compact Discs

| **Frequency of Use** | **Frequency** | **Percent** | **Valid Percent** | **Cumulative Percent** |
| --- | --- | --- | --- | --- |
| Never Used | 19 | 25.3 | 25.3 | 25.3 |
| Used Sometimes | 34 | 45.3 | 45.3 | 70.7 |
| Used Always | 22 | 29.3 | 29.3 | 100.0 |
| Total | 75 | 100.0 | 100.0 |  |

.

**Table 13:** Voice over Internet Protocol (e.g. Skype)

| **Frequency of Use** | Frequency | Percent | Valid Percent | Cumulative Percent |
| --- | --- | --- | --- | --- |
| Never Used | 14 | 18.7 | 18.7 | 18.7 |
| Used Sometimes | 29 | 38.7 | 38.7 | 57.3 |
| Used Always | 32 | 42.7 | 42.7 | 100.0 |
| Total | 75 | 100.0 | 100.0 |  |

**Table 14: 3-D printer**

| **Frequency of Use** | **Frequency** | **Percent** | **Valid Percent** | **Cumulative Percent** |
| --- | --- | --- | --- | --- |
| Never Used | 27 | 36.0 | 36.0 | 36.0 |
| Used Sometimes | 30 | 40.0 | 40.0 | 76.0 |
| Used Always | 18 | 24.0 | 24.0 | 100.0 |
| Total | 75 | 100.0 | 100.0 |  |

**Frequency of use of the digital technologies at the tendering stage of building projects**

**Table 15: Web-based Enterprise Resource Planning**

| **Frequency of Use** | **Frequency** | **Percent** | **Valid Percent** | **Cumulative Percent** |
| --- | --- | --- | --- | --- |
| Never Used | 31 | 41.3 | 41.3 | 41.3 |
| Used Sometimes | 34 | 45.3 | 45.3 | 86.7 |
| Used Always | 10 | 13.3 | 13.3 | 100.0 |
| **Total** | **75** | **100.0** | **100.0** |  |

**Table 16: Web-based Project Portals**

| **Frequency of Use** | **Frequency** | **Percent** | **Valid Percent** | **Cumulative Percent** |
| --- | --- | --- | --- | --- |
| **Never Used** | 17 | 22.7 | 22.7 | 22.7 |
| **Used Sometimes** | 44 | 58.7 | 58.7 | 81.3 |
| **Used Always** | 14 | 18.7 | 18.7 | 100.0 |
| **Total** | **75** | **100.0** | **100.0** |  |

**Table 17: Cloud Computing Technology (CCT)**

| **Frequency of Use** | Frequency | Percent | Valid Percent | Cumulative Percent |
| --- | --- | --- | --- | --- |
| **Never Used** | 15 | 20.0 | 20.0 | 20.0 |
| **Used Sometimes** | 41 | 54.7 | 54.7 | 74.7 |
| **Used Always** | 19 | 25.3 | 25.3 | 100.0 |
| **Total** | **75** | **100.0** | **100.0** |  |

**Table 18: Teleconferencing Technology**

| **Frequency of Use** | **Frequency** | **Percent** | **Valid Percent** | **Cumulative Percent** |
| --- | --- | --- | --- | --- |
| **Never Used** | 16 | 21.3 | 21.3 | 21.3 |
| **Used Sometimes** | 28 | 37.3 | 37.3 | 58.7 |
| **Used Always** | 31 | 41.3 | 41.3 | 100.0 |
| **Total** | **75** | **100.0** | **100.0** |  |

**Table 19: Web-Based Project management software packages**

| **Frequency of Use** | Frequency | Percent | Valid Percent | Cumulative Percent |
| --- | --- | --- | --- | --- |
| Never Used | 16 | 21.3 | 21.3 | 21.3 |
| Used Sometimes | 39 | 52.0 | 52.0 | 73.3 |
| Used Always | 20 | 26.7 | 26.7 | 100.0 |
| **Total** | **75** | **100.0** | **100.0** |  |

**Table 20:** Drop box

| **Frequency of Use** | **Frequency** | **Percent** | **Valid Percent** | **Cumulative Percent** |
| --- | --- | --- | --- | --- |
| **Never Used** | 15 | 20.0 | 20.0 | 20.0 |
| **Used Sometimes** | 31 | 41.3 | 41.3 | 61.3 |
| **Used Always** | 29 | 38.7 | 38.7 | 100.0 |
| **Total** | 75 | 100.0 | 100.0 |  |

**Table 21: Microsoft SharePoint**

| **Frequency of Use** | **Frequency** | **Percent** | **Valid Percent** | **Cumulative Percent** |
| --- | --- | --- | --- | --- |
| Never Used | 30 | 40.0 | 40.0 | 40.0 |
| Used Sometimes | 26 | 34.7 | 34.7 | 74.7 |
| Used Always | 19 | 25.3 | 25.3 | 100.0 |
| **Total** | **75** | **100.0** | **100.0** |  |

**Table 22**: Google Documents

| **Frequency of Use** | **Frequency** | **Percent** | **Valid Percent** | **Cumulative Percent** |
| --- | --- | --- | --- | --- |
| **Never Used** | 15 | 20.0 | 20.0 | 20.0 |
| **Used Sometimes** | 32 | 42.7 | 42.7 | 62.7 |
| **Used Always** | 28 | 37.3 | 37.3 | 100.0 |
| **Total** | 75 | 100.0 | 100.0 |  |

.

**Figure 4: Network technologies**

**Table 23**: Frequency of Use of Fax

| **Frequency of Use** | **Frequency** | **Percent** | **Valid Percent** | **Cumulative Percent** |
| --- | --- | --- | --- | --- |
| **Never Used** | 27 | 36.0 | 36.0 | 36.0 |
| **Used Sometimes** | 28 | 37.3 | 37.3 | 73.3 |
| **Used Always** | 20 | 26.7 | 26.7 | 100.0 |
| **Total** | **75** | **100.0** | **100.0** |  |

**Frequency of use of the digital technologies at the construction stage of building projects**

**Table 24: Project camera**

| **Frequency of Use** | **Frequency** | **Percent** | **Valid Percent** | **Cumulative Percent** |
| --- | --- | --- | --- | --- |
| **Never Used** | 8 | 10.7 | 10.7 | 10.8 |
| **Used Sometimes** | 31 | 41.3 | 41.9 | 52.7 |
| **Used Always** | 35 | 46.7 | 47.3 | 100.0 |
| **No Response** | 1 | 1.3 | 1.3 |  |
| **Total** | 75 | 100.0 | 100.0 |  |

**Table 25: Bar Code technology**

| **Frequency of Use** | **Frequency** | **Percent** | **Valid Percent** | **Cumulative Percent** |
| --- | --- | --- | --- | --- |
| **Never Used** | 38 | 50.7 | 50.7 | 50.7 |
| **Used Sometimes** | 23 | 30.7 | 30.7 | 81.3 |
| **Used Always** | 14 | 18.7 | 18.7 | 100.0 |
| **Total** | **75** | **100.0** | **100.0** |  |

**Table 26**: Radio Frequency Identification (RFID)

| **Frequency of Use** | | **Frequency** | | **Percent** | | **Valid Percent** | | **Cumulative Percent** | |
| --- | --- | --- | --- | --- | --- | --- | --- | --- | --- |
| **Never Used** | 36 | | 48.0 | | 48.0 | | 48.0 | |  |
| **Used Sometimes** | 24 | | 32.0 | | 32.0 | | 80.0 | |  |
| **Used Always** | 15 | | 20.0 | | 20.0 | | 100.0 | |  |
| **Total** | 75 | | 100.0 | | 100.0 | |  | |  |

**Table 27**: Electronic Data Interchange (EDI)

| **Frequency of Use** | **Frequency** | **Percent** | **Valid Percent** | **Cumulative Percent** |
| --- | --- | --- | --- | --- |
| **Never Used** | 26 | 34.7 | 34.7 | 34.7 |
| **Used Sometimes** | 17 | 22.7 | 22.7 | 57.3 |
| **Used Always** | 32 | 42.7 | 42.7 | 100.0 |
| **Total** | 75 | 100.0 | 100.0 |  |

**Figure 5: E-Marketplaces**

**Table 28: Internet-Based Geographic Information System (GIS)**

| **Frequency of Use** | **Frequency** | **Percent** | **Valid Percent** | **Cumulative Percent** |
| --- | --- | --- | --- | --- |
| **Never Used** | 21 | 28.0 | 28.0 | 28.0 |
| **Used Sometimes** | 29 | 38.7 | 38.7 | 66.7 |
| **Used Always** | 25 | 33.3 | 33.3 | 100.0 |
| **Total** | **75** | **100.0** | **100.0** |  |

**Table 29: Geographic Positioning System (GPS)**

| **Frequency of Use** | **Frequency** | **Percent** | **Valid Percent** | **Cumulative Percent** |
| --- | --- | --- | --- | --- |
| **Never Used** | 11 | 14.7 | 14.7 | 14.7 |
| **Used Sometimes** | 35 | 46.7 | 46.7 | 61.3 |
| **Used Always** | 29 | 38.7 | 38.7 | 100.0 |
| **Total** | **75** | **100.0** | **100.0** |  |

**Table 30: Web-based Project Portals**

| **Frequency of Use** | **Frequency** | **Percent** | **Valid Percent** | **Cumulative Percent** |
| --- | --- | --- | --- | --- |
| **Never Used** | 19 | 25.3 | 25.3 | 25.3 |
| **Used Sometimes** | 39 | 52.0 | 52.0 | 77.3 |
| **Used Always** | 17 | 22.7 | 22.7 | 100.0 |
| **Total** | **75** | **100.0** | **100.0** |  |

**Figure 6: Wireless Technology (WT)**

**Figure 7: Web.2.0 technology (e.g. social media platforms)**

**Figure 8: Use of Computers**

.

**Figure 9: The Use of E-mail technology**

**Figure 10: The use of Telephones**

**Figure 11: Building Information Modelling (BIM)**

**Advantages of the use of digital technologies in building	project delivery**

**Figure 12: Access to centrally stored information**

**Figure 13: Saves times**

**Figure 14: Saves cost of project delivery**

**Figure 15: Reduces the use of paper work**

**Figure 16: Makes it easier exchange of project information**

**Figure 17: Helps in monitoring project site and collecting real-time data**

**Figure 18: Promotes collaborative work**

**Figure 19: Helps in inventory management and record keeping**

**Figure 20: Promotes integration of tasks among consultants**

**Figure 21: Automates Project Delivery Process**

**Figure 22: Facilitates the reduction of waste in project delivery**

.

**Figure 23: Promotes efficiency in project delivery**

.

**Figure 24: Improves Productivity of firms**

**Table 31**: The Use of DTs is less labour intensive

|  | **Frequency** | **Percent** | **Valid Percent** | **Cumulative Percent** |
| --- | --- | --- | --- | --- |
| **Strongly Disagree** | 3 | 4.0 | 4.0 | 4.0 |
| **Disagree** | 5 | 6.7 | 6.7 | 10.7 |
| **Undecided** | 8 | 10.7 | 10.7 | 21.3 |
| **Agree** | 31 | 41.3 | 41.3 | 62.7 |
| **Strongly Agree** | 28 | 37.3 | 37.3 | 100.0 |
| **Total** | 75 | 100.0 | 100.0 |  |

.

**Table 36: Reduces Errors**

|  | **Frequency** | **Percent** | **Valid Percent** | **Cumulative Percent** |
| --- | --- | --- | --- | --- |
| **Strongly Disagree** | 4 | 5.3 | 5.3 | 5.3 |
| **Disagree** | 7 | 9.3 | 9.3 | 14.7 |
| **Undecided** | 8 | 10.7 | 10.7 | 25.3 |
| **Agree** | 30 | 40.0 | 40.0 | 65.3 |
| **Strongly Agree** | 26 | 34.7 | 34.7 | 100.0 |
| **Total** | 75 | 100.0 | 100.0 |  |

**Source:** Field Work, (2018)

 **Figure 25: Facilitates quick decision making**

**Table	33: Elimination of Geographic barrier in project execution**

|  | **Frequency** | **Percent** | **Valid Percent** | **Cumulative Percent** |
| --- | --- | --- | --- | --- |
| **Strongly Disagree** | 1 | 1.3 | 1.3 | 1.3 |
| **Disagree** | 3 | 4.0 | 4.0 | 5.3 |
| **Undecided** | 10 | 13.3 | 13.3 | 18.7 |
| **Agree** | 30 | 40.0 | 40.0 | 58.7 |
| **Strongly Agree** | 31 | 41.3 | 41.3 | 100.0 |
| **Total** | 75 | 100.0 | 100.0 |  |

**Table 34: Descriptive Statistics of the Advantages of DTs in Project Delivery**

| **Advantages** | **Mean** | **Standard deviation** | **Rank** |
| --- | --- | --- | --- |
| Saves time in project delivery | 4.28 | 1.021 | 1 |
| Improves productivity of firms | 4.25 | 0.931 | 2 |
| Saves cost in project delivery | 4.24 | 0.970 | 3 |
| Reduces the use of paper work | 4.24 | 0.998 | 3 |
| Allows for access to centrally stored information | 4.19 | 1.009 | 5 |
| Elimination of geographic barrier in project execution | 4.16 | 0.901 | 6 |
| Automates project delivery process | 4.15 | 0.881 | 7 |
| Promotes collaborative work | 4.13 | 0.949 | 8 |
| Helps in inventory management and record keeping | 4.13 | 0.935 | 8 |
| Facilitates the reduction of waste in project delivery | 4.12 | 1.090 | 10 |
| Makes it easier exchange of project information | 4.11 | 1.098 | 11 |
| Promotes efficiency in project delivery | 4.07 | 0.977 | 12 |
| Promotes integration of tasks among consultants | 4.05 | 0.928 | 13 |
| Is less labour intensive | 4.01 | 1.059 | 14 |
| Facilitates quick decision making | 3.96 | 1.108 | 15 |
| Helps in monitoring project site and collect real-time data | 3.95 | 1.196 | 16 |
| Reduces errors | 3.89 | 1.146 | 17 |

**Challenges in the use of Digital Technologies in Building Project Delivery**

**Figure 26: Cost of acquiring and use of digital technologies**

.

**Table 35:** Lack of Confidentiality of Project Data

|  | **Frequency** | **Percent** | **Valid Percent** | **Cumulative Percent** |
| --- | --- | --- | --- | --- |
| Strongly Disagree | 9 | 12.0 | 12.0 | 12.0 |
| Disagree | 17 | 22.7 | 22.7 | 34.7 |
| Undecided | 8 | 10.7 | 10.7 | 45.3 |
| Agree | 35 | 46.7 | 46.7 | 92.0 |
| Strongly Agree | 6 | 8.0 | 8.0 | 100.0 |
| Total | 75 | 100.0 | 100.0 |  |

**Table 36:** Problem of incompatibility of different applications

|  | Frequency | Percent | Valid Percent | Cumulative Percent |
| --- | --- | --- | --- | --- |
| Strongly Disagree | 5 | 6.7 | 6.7 | 6.7 |
| Disagree | 8 | 10.7 | 10.7 | 17.3 |
| Undecided | 10 | 13.3 | 13.3 | 30.7 |
| Agree | 44 | 58.7 | 58.7 | 89.3 |
| Strongly Agree | 8 | 10.7 | 10.7 | 100.0 |
| Total | 75 | 100.0 | 100.0 |  |

**Table 37: Some Digital Technologies are not Available in Nigeria**

|  | **Frequency** | **Percent** | **Valid Percent** | **Cumulative Percent** |
| --- | --- | --- | --- | --- |
| **Strongly Disagree** | 2 | 2.7 | 2.7 | 2.7 |
| **Disagree** | 4 | 5.3 | 5.3 | 8.0 |
| **Undecided** | 17 | 22.7 | 22.7 | 30.7 |
| **Agree** | 31 | 41.3 | 41.3 | 72.0 |
| **Strongly Agree** | 21 | 28.0 | 28.0 | 100.0 |
| **Total** | 75 | 100.0 | 100.0 |  |

**Table 38:** Special training is required before one can use digital technologies

|  | **Frequency** | **Percent** | **Valid Percent** | **Cumulative Percent** |
| --- | --- | --- | --- | --- |
| **Strongly Disagree** | 4 | 5.3 | 5.3 | 5.3 |
| **Disagree** | 6 | 8.0 | 8.0 | 13.3 |
| **Undecided** | 11 | 14.7 | 14.7 | 28.0 |
| **Agree** | 38 | 50.7 | 50.7 | 78.7 |
| **Strongly Agree** | 16 | 21.3 | 21.3 | 100.0 |
| **Total** | 75 | 100.0 | 100.0 |  |

**Figure 26: Many people in the building industry do not know about DTs**

**Table 39:** Delays in the Transmission of Data and Information via the Internet

|  | **Frequency** | **Percent** | **Valid Percent** | **Cumulative Percent** |
| --- | --- | --- | --- | --- |
| **Strongly Disagree** | 8 | 10.7 | 10.7 | 10.7 |
| **Disagree** | 15 | 20.0 | 20.0 | 30.7 |
| **Undecided** | 10 | 13.3 | 13.3 | 44.0 |
| **Agree** | 30 | 40.0 | 40.0 | 84.0 |
| **Strongly Agree** | 12 | 16.0 | 16.0 | 100.0 |
| **Total** | 75 | 100.0 | 100.0 |  |

**Figure 27: DTs do not Encourage Creativity in Architectural Design**

**Table 40:** Digital Technologies Use can lead to job loss in the Industry

|  | **Frequency** | **Percent** | **Valid Percent** | **Cumulative Percent** |
| --- | --- | --- | --- | --- |
| Strongly Disagree | 8 | 10.7 | 10.7 | 10.7 |
| Disagree | 5 | 6.7 | 6.7 | 17.3 |
| Undecided | 14 | 18.7 | 18.7 | 36.0 |
| Agree | 31 | 41.3 | 41.3 | 77.3 |
| Strongly Agree | 17 | 22.7 | 22.7 | 100.0 |
| Total | 75 | 100.0 | 100.0 |  |

**Figure 28: Insecurity of data with digital technologies**

**Some digital technologies are not user friendly**

|  | Frequency | Percent | Valid Percent | Cumulative Percent |
| --- | --- | --- | --- | --- |
| Strongly Disagree | 2 | 2.7 | 2.7 | 2.7 |
| Disagree | 4 | 5.3 | 5.3 | 8.0 |
| Undecided | 17 | 22.7 | 22.7 | 30.7 |
| Agree | 31 | 41.3 | 41.3 | 72.0 |

**Table 41: Descriptive Statistics of Challenges of using of Digital Technologies in Building Project Delivery**

| **Challenges** | **Mean** | **Standard deviation** | **Rank** |
| --- | --- | --- | --- |
| Digital technologies are expensive to acquire and use | 3.95 | 0.928 | 1 |
| Some digital technologies are not available in Nigeria | 3.87 | 0.977 | 2 |
| Special training is required before one can use digital technologies | 3.75 | 1.054 | 3 |
| The use of digital technologies can lead to job loss in the industry | 3.59 | 1.220 | 4 |
| Some digital technologies are not user friendly | 3.57 | 1.068 | 5 |
| There is the problem of incompatibility of different applications | 3.56 | 1.043 | 6 |
| Many people in the building industry do not know about digital technologies | 3.52 | 1.119 | 7 |
| There are delays in the transmission of data and information via the internet | 3.31 | 1.262 | 8 |
| There is insecurity of data with digital technologies | 3.21 | 1.244 | 9 |
| There is lack of confidentiality of project data | 3.16 | 1.220 | 10 |
| Some digital technologies do not encourage creativity in architectural design | 2.92 | 1.383 | 11 |
